# Transcriptome of *Sphaerospora molnari* (Cnidaria, Myxosporea) blood stages provides proteolytic arsenal as potential therapeutic targets against sphaerosporosis in common carp

**DOI:** 10.1186/s12864-020-6705-y

**Published:** 2020-06-16

**Authors:** Ashlie Hartigan, Anush Kosakyan, Hana Pecková, Edit Eszterbauer, Astrid S. Holzer

**Affiliations:** 1 Institute of Parasitology, Biology Centre, Czech Academy of Science, České Budějovice, Czechia; 2 Institute for Veterinary Medical Research, Centre for Agricultural Research, Hungarian Academy of Sciences, Budapest, Hungary

**Keywords:** Myxozoa, In silico screening, Proteases, Aquaculture, Parasite, Drug targets

## Abstract

**Background:**

Parasites employ proteases to evade host immune systems, feed and replicate and are often the target of anti-parasite strategies to disrupt these interactions. Myxozoans are obligate cnidarian parasites, alternating between invertebrate and fish hosts. Their genes are highly divergent from other metazoans, and available genomic and transcriptomic datasets are limited. Some myxozoans are important aquaculture pathogens such as *Sphaerospora molnari* replicating in the blood of farmed carp before reaching the gills for sporogenesis and transmission. Proliferative stages cause a massive systemic lymphocyte response and the disruption of the gill epithelia by spore-forming stages leads to respiratory problems and mortalities. In the absence of a *S. molnari* genome, we utilized a de novo approach to assemble the first transcriptome of proliferative myxozoan stages to identify *S. molnari* proteases that are upregulated during the first stages of infection when the parasite multiplies massively, rather than in late spore-forming plasmodia. Furthermore, a subset of orthologs was used to characterize 3D structures and putative druggable targets.

**Results:**

An assembled and host filtered transcriptome containing 9436 proteins, mapping to 29,560 contigs was mined for protease virulence factors and revealed that cysteine proteases were most common (38%), at a higher percentage than other myxozoans or cnidarians (25–30%). Two cathepsin Ls that were found upregulated in spore-forming stages with a presenilin like aspartic protease and a dipeptidyl peptidase. We also identified downregulated proteases in the spore-forming development when compared with proliferative stages including an astacin metallopeptidase and lipases (qPCR). In total, 235 transcripts were identified as putative proteases using a MEROPS database. In silico analysis of highly transcribed cathepsins revealed potential drug targets within this data set that should be prioritised for development.

**Conclusions:**

In silico surveys for proteins are essential in drug discovery and understanding host-parasite interactions in non-model systems. The present study of *S. molnari*’s protease arsenal reveals previously unknown proteases potentially used for host exploitation and immune evasion. The pioneering dataset serves as a model for myxozoan virulence research, which is of particular importance as myxozoan diseases have recently been shown to emerge and expand geographically, due to climate change.

## Background

The relationship between parasites and their hosts is under constant pressure; parasites must invade, replicate and feed whilst avoiding the host immune system. Proteases are the weapon of choice for parasites to overcome these challenges within the host, and can be specifically adapted for cleaving host proteins or modifying their own proteins for immune system avoidance [1–3]. Proteases are often high priority proteins for investigation as they have essential roles in development, invasion or feeding [4]. However, proteases are involved in other cellular functions e.g. transport and activation of other peptidases, and it can often be unclear which peptidases are essential to parasite survival or success [5, 6]. Drug or interference targets can be difficult to identify in a wash of uncharacterized proteins, however proteases linked to an essential cellular pathway or localised to a particular organelle e.g. lysosome can be considered useful targets for life cycle or development disruption [5, 7].

Anti-parasite drugs currently available have been identified by screening sets of compounds in vitro culture systems and by borrowing compounds that have worked in other pathogens and applying those to a new parasite model [8]. Firstly, this limits progress to organisms and life stages that can be isolated and cultured; secondly it relies on applicable compounds having been found in related organisms; and thirdly it limits discovery as it looks at one target at a time for feasibility. In silico drug target discovery in contrast has the attractive attributes of speed, low cost and no requirement for living parasites. In the case of non-model organisms this is likely the first step before prioritising any protein for further experimentation with the aim of anti-parasite treatment development.

Myxozoans are parasitic cnidarians that are important pathogens to both wild and cultured fish populations and yet there are no drug targets specified for this group and limited proteolytic studies to examine activity or function of selected proteins [9, 10]. Myxozoans are suggested to have reduced genomes compared to their free living cnidarian relatives [11, 12] which could have an impact on the range and diversity of the peptidases expressed. Many aspects of myxozoan biology are still unknown or inferred by comparison with other parasites to infer biology such as their metabolism (*Thelohanellus kitauei* - [12]), their replication [13] or proteins interacting with the host immune system (reviewed in [14]). Myxozoans are entirely parasitic in their life cycle, they alternate between a vertebrate and an invertebrate host with two entirely different types of transmissible spores in each developmental phase [15–18]. Myxospores are often hardy stages that are capable of being exposed to the environment for long periods of time waiting for uptake by their invertebrate hosts. The actinospores are generally more fragile and only viable for a limited period of time as they are released into the water column to encounter a suitable vertebrate host [19]. There are two main sources of material for genomic and transcriptomic analysis, plasmodia or cysts of developing myxospores from the vertebrate [11, 12] or actinospores released from their invertebrate host [11]. Spore development represents the final step prior to transmission with the genetic arsenal related to their production of durable spores often expressed in cysts, separated from the host immune response by connective tissue, while actinospores are collected from the environment, prior to infecting their vertebrate hosts. Therefore, they do not provide many insights into what proteins are helping the parasite feed or replicate or evade immune detection.

**Fig. 1:**
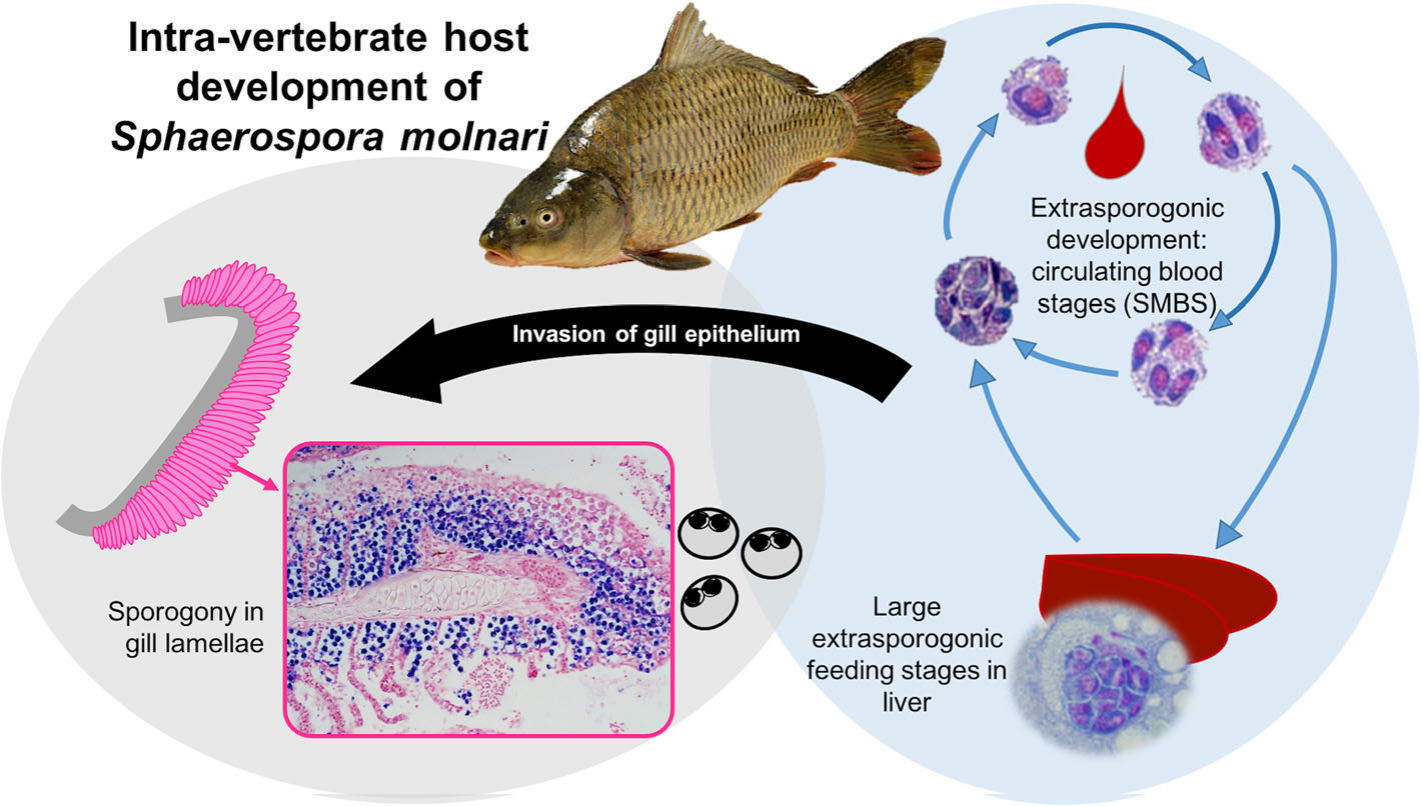
Developmental cycle of *Sphaerospora molnari* within its host *Cyprinus carpio*. *Sphaerospora molnari* blood stages and infected gill images by A.S. Holzer, (blood stages modified from [28, 29]). Common carp (host) royalty free stock image (dreamstime.com)

Sphaerosporids are a major clade of the Myxosporea, with a large proportion found in bony and cartilaginous fish, and amphibians [20–24]. A specific trait that has only been identified in this clade is the presence of large, extracellular stages circulating in the blood stream of their fish hosts [25–27]. The parasites not only use the blood for transport to their target organ but proliferate within it and are present almost all year round (Fig. 1, [26, 28, 30]). *Sphaerospora molnari* is a parasite of the common carp in Central Europe with motile blood stages that provoke a strong immune response [29] and are a likely co-factor for developing Swim Bladder Inflammation [30]. *S. molnari* blood stages (SMBS) are prime targets for parasite intervention therapy, as they are 1) responsible for massive proliferation in the earliest stages of infection of fish, 2) freely circulating in the blood and any drug targeting the SMBS would not need to be applied to host tissue or taken up by host cells; 3) they are circulating in the blood for an extended period and therefore there is a longer window for application of anti-parasite therapies. In addition, preliminary protein studies on SMBS show a high level of sequence divergence even in highly conserved proteins such as actin [28] and therefore SMBS could potentially have proteases that are highly divergent from their hosts as well as other cnidarians which would aid protein target assay development. This study examines protease families and groups present in the transcriptome of SMBS to investigate their diversity and divergence. We compare key protease groups with examples known from other parasites that have been successfully flagged as drug or anti-parasite targets. In addition, we provide gene expression data for selected candidates with the goal of identifying stage-specific proteases of interest for future functional studies.

## Results

This transcriptome is the first next generation sequencing dataset from any sphaerosporid species, and also the first dataset from a highly proliferative, extrasporogonic developmental myxozoan stage. Pooled host (*Cyprinus carpio*) blood cells and *Sphaerospora molnari* blood stages (SMBS) from 8 infected fish were used for this transcriptome. Illumina HiSeq sequencing yielded a total of 52,040,447 clean paired reads, mapping these to the gene models of *C. carpio* removed 14,849,448 reads (BioProject PRJNA522909). A trinity assembly of these remaining reads gave 157,506 transcripts, (mean length 766 nt, 39.75% GC content). 127,741 of these transcripts (81.1%) were found in the carp genome with an e-value of 1e^− 05^ or more and a percentage identity of > 75%. The remaining 29,765 transcripts were therefore assumed to be *S. molnari*, and were translated into 29,588 proteins (Table 1). To examine the presence of potential chimeric sequences, we amplified a substantial number (*n* = 15) of our flagged proteases and ribosomal DNA to verify their sequence according to our assembly. Sanger sequencing of the complete ribosomal DNA yielded 13,486 bp (Genbank Acc. Nr MK533682), and large fragments of all flagged proteases were also verified from cDNA (Supplementary Data).

**Table 1:** Summary of *S. molnari* blood stages (SMBS) transcriptome dataset

Sequence dataset	Number of sequences
Trinity output (post read filtering)	157,506
Total transcriptome GC content	39.8%
Transcripts matching carp genome	127, 741
Carp transcripts GC content	40.9%
Sequences not matching to carp genome	29,765
GC content	35.7%
Non-carp transcripts with ORF	29,588
No. of proteins annotated with nr	9436
Peptidase candidates	235

Screening with BUSCO, identified that *S. molnari* retained and is expressing at least half of the 978 benchmark metazoan genes [31] (53% of the single copy metazoan genes). We completed the same analysis for myxozoans *Myxobolus cerebralis* and *Kudoa iwatai*, and non-myxozoans *Polypodium hydriforme*, *Edwardsiella lineata*, *Nematostella vectensis* (Table 2). *S. molnari* had the highest number of complete BUSCOs of all the myxozoan datasets, whereas *N. vectensis* had the highest overall (905/978, 93%), similar results were found for the single copy BUSCOs. *S. molnari* had the lowest number of missing genes (374/978, 38.2%) within the myxozoans, in comparison *N. vectensis* was only missing 30 genes (30/978, 3%) (Table 2).

**Table 2:** Benchmarking Universal Single Copy Orthologs (BUSCO) identified in datasets

	***S. molnari***	***M. cerebralis***	***K. iwatai***	***P. hydriforme***	***E. lineata***	***N. vectensis***
**Complete BUSCOS**	551	484	471	842	599	905
**Complete single cp BUSCO**	521	287	302	815	578	888
**Frag BUSCO**	53	60	78	30	286	43
**Missing BUSCO**	374	434	429	106	93	30

**Fig. 2:**
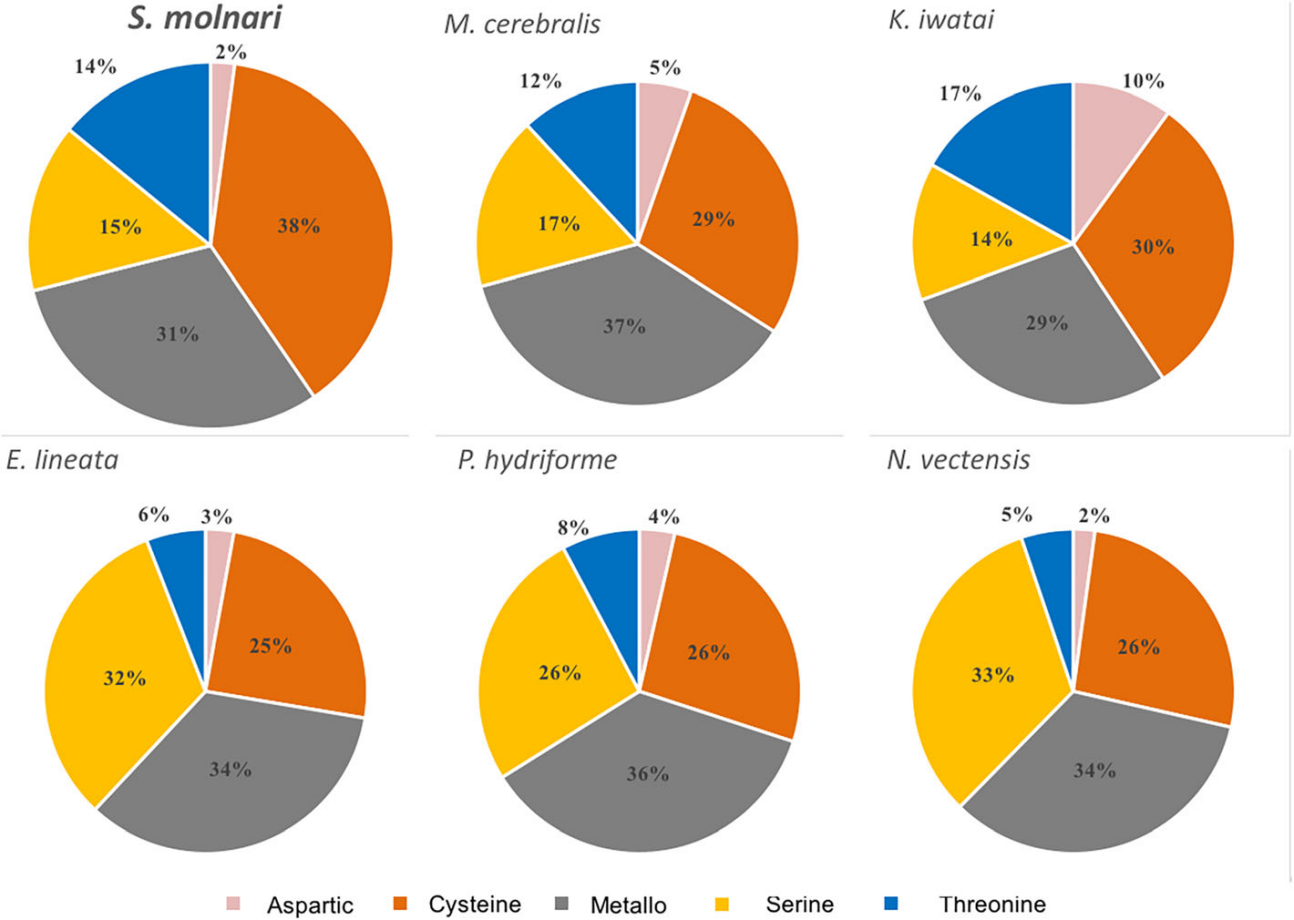
Comparative summary of expressed proteases identified in three myxozoan datasets, two non-myxozoan parasitic cnidarian datasets and a free living cnidarian species. Pie charts showing diversity and abundance of protease clans and families in transcriptomes of *Sphaerospora molnari* and other myxozoans (*Myxobolus cerebralis* and *Kudoa iwatai*), two non-myxozoan parasitic cnidarians (*Edwardsiella lineata* and *Polypodium hydriforme*) and free living species *Nematostella vectensis*

We queried the transcriptomic dataset of SMBS for proteases using representative protease sequences downloaded from the MEROPS database. Less than 1% of all transcripts had a strong sequence match. There were 235 homologs identified in SMBS representing 45 peptidase families, the majority of the proteases were cysteine (38%), followed by metalloproteases (31%), serine (15%), threonine (14%) and aspartic groups (2%) (Fig. 2). Families that were highly represented in SMBS (Table 3) were C01 Papain-like proteases, C12 and C19 – ubiquitinyl hydrolases, M24 – aminopeptidases and dipeptidyl peptidases, M67 – ubiquitin releasing proteases often associated with proteasomal degradation and T01 – proteasome proteases. There were many families that were absent in all examined cnidarians, and even more, missing from only the myxozoans compared to the free living species e.g. S28 lysosomal carboxypeptidase.

**Table 3:** Protease families identified in *S. molnari* bood stages (SMBS) and other cnidarian species

Clan	Family	***S. molnari***	***M. cerebralis***	***K. iwatai***	***P. hydriforme***	***E. lineata***	***N. vectensis***
**Aspartic**	**A01**	**2**	**4**	**13**	**4**	**3**	**3**
	**A02**	**0**	**0**	**0**	**0**	**0**	**0**
	**A08**	**0**	**0**	**0**	**0**	**0**	**1**
	**A11**	**0**	**0**	**0**	**0**	**1**	**0**
	**A20**	**0**	**0**	**0**	**0**	**1**	**0**
	**A22**	**3**	**4**	**3**	**5**	**6**	**4**
	**A24**	**0**	**0**	**0**	**0**	**0**	**0**
	**A28**	**0**	**2**	**0**	**1**	**1**	**2**
**Cysteine**	**C01**	**18**	**10**	**12**	**7**	**13**	**14**
	**C02**	**0**	**0**	**0**	**5**	**6**	**6**
	**C12**	**8**	**3**	**4**	**3**	**3**	**3**
	**C13**	**0**	**0**	**0**	**2**	**2**	**2**
	**C14**	**0**	**1**	**0**	**4**	**6**	**5**
	**C15**	**0**	**0**	**0**	**0**	**1**	**1**
	**C19**	**34**	**18**	**20**	**18**	**26**	**30**
	**C26**	**3**	**2**	**0**	**5**	**7**	**11**
	**C39**	**0**	**0**	**0**	**0**	**0**	**0**
	**C40**	**0**	**0**	**0**	**0**	**0**	**0**
	**C44**	**2**	**1**	**1**	**2**	**5**	**6**
	**C46**	**0**	**0**	**0**	**1**	**2**	**5**
	**C48**	**7**	**4**	**7**	**5**	**3**	**5**
	**C50**	**2**	**2**	**1**	**1**	**1**	**1**
	**C54**	**3**	**1**	**3**	**3**	**2**	**2**
	**C56**	**3**	**2**	**1**	**4**	**5**	**4**
	**C64**	**0**	**0**	**0**	**1**	**2**	**4**
	**C65**	**1**	**0**	**0**	**1**	**1**	**1**
	**C67**	**0**	**0**	**0**	**0**	**2**	**1**
	**C69**	**0**	**0**	**0**	**0**	**0**	**0**
	**C78**	**1**	**2**	**0**	**1**	**1**	**2**
	**C83**	**0**	**0**	**0**	**0**	**0**	**1**
	**C85**	**5**	**2**	**0**	**3**	**5**	**8**
	**C86**	**0**	**1**	**0**	**2**	**2**	**0**
	**C89**	**1**	**0**	**0**	**2**	**2**	**2**
	**C93**	**0**	**0**	**0**	**0**	**0**	**0**
	**C95**	**0**	**0**	**0**	**1**	**2**	**2**
	**C97**	**1**	**2**	**0**	**2**	**2**	**2**
	**C98**	**0**	**0**	**0**	**1**	**1**	**1**
	**C101**	**0**	**0**	**0**	**0**	**0**	**0**
	**C110**	**0**	**0**	**0**	**0**	**1**	**1**
	**C111**	**1**	**1**	**0**	**0**	**1**	**1**
	**C115**	**0**	**1**	**0**	**1**	**1**	**1**
**Metallo**	**M01**	**3**	**3**	**2**	**9**	**10**	**9**
	**M02**	**0**	**0**	**0**	**2**	**3**	**2**
	**M03**	**4**	**4**	**4**	**1**	**3**	**4**
	**M08**	**0**	**0**	**0**	**1**	**1**	**2**
	**M10**	**0**	**0**	**0**	**4**	**6**	**9**
	**M12**	**17**	**22**	**7**	**26**	**34**	**30**
	**M13**	**2**	**3**	**2**	**6**	**11**	**10**
	**M14**	**1**	**6**	**4**	**5**	**13**	**12**
	**M16**	**10**	**10**	**5**	**9**	**10**	**8**
	**M17**	**4**	**2**	**1**	**2**	**3**	**4**
	**M18**	**0**	**1**	**1**	**1**	**1**	**1**
	**M19**	**0**	**0**	**0**	**0**	**1**	**2**
	**M20**	**2**	**2**	**1**	**3**	**5**	**6**
	**M24**	**10**	**5**	**6**	**8**	**8**	**10**
	**M28**	**2**	**1**	**2**	**6**	**11**	**13**
	**M38**	**1**	**0**	**1**	**3**	**5**	**10**
	**M41**	**3**	**1**	**4**	**3**	**3**	**3**
	**M43**	**0**	**0**	**0**	**0**	**1**	**2**
	**M48**	**1**	**0**	**2**	**1**	**2**	**3**
	**M49**	**0**	**0**	**0**	**0**	**1**	**0**
	**M50**	**0**	**0**	**0**	**2**	**1**	**1**
	**M54**	**0**	**0**	**0**	**0**	**1**	**2**
	**M61**	**0**	**0**	**0**	**0**	**0**	**0**
	**M67**	**10**	**6**	**3**	**8**	**9**	**10**
	**M76**	**0**	**0**	**0**	**0**	**1**	**1**
	**M79**	**1**	**1**	**1**	**1**	**0**	**1**
	**M96**	**0**	**0**	**0**	**0**	**1**	**1**
	**M98**	**0**	**0**	**0**	**0**	**0**	**0**
	**M100**	**1**	**1**	**0**	**1**	**0**	**0**
**Mixed**	**P01**	**0**	**0**	**0**	**0**	**0**	**0**
	**P02**	**0**	**0**	**0**	**9**	**15**	**12**
**Serine**	**S01**	**3**	**1**	**0**	**23**	**53**	**54**
	**S08**	**2**	**6**	**5**	**7**	**8**	**12**
	**S09**	**13**	**10**	**8**	**11**	**27**	**32**
	**S10**	**0**	**0**	**0**	**0**	**2**	**1**
	**S11**	**0**	**0**	**0**	**0**	**0**	**0**
	**S12**	**2**	**0**	**2**	**0**	**5**	**4**
	**S13**	**0**	**0**	**0**	**0**	**0**	**0**
	**S14**	**0**	**0**	**0**	**1**	**1**	**1**
	**S16**	**1**	**1**	**0**	**1**	**2**	**2**
	**S24**	**0**	**0**	**0**	**0**	**0**	**1**
	**S26**	**3**	**3**	**3**	**3**	**3**	**3**
	**S28**	**0**	**0**	**0**	**3**	**5**	**4**
	**S33**	**9**	**7**	**1**	**18**	**20**	**23**
	**S41**	**0**	**0**	**0**	**0**	**0**	**2**
	**S49**	**0**	**0**	**0**	**0**	**1**	**2**
	**S50**	**0**	**0**	**0**	**0**	**0**	**0**
	**S53**	**0**	**0**	**0**	**1**	**1**	**1**
	**S54**	**1**	**1**	**1**	**3**	**3**	**4**
	**S59**	**1**	**3**	**2**	**1**	**1**	**1**
	**S60**	**0**	**0**	**0**	**2**	**1**	**1**
	**S68**	**0**	**0**	**0**	**0**	**0**	**0**
	**S71**	**0**	**0**	**0**	**0**	**1**	**1**
	**S72**	**0**	**0**	**0**	**0**	**2**	**1**
	**S79**	**0**	**0**	**0**	**0**	**0**	**0**
**Threonine**	**T01**	**22**	**14**	**11**	**14**	**15**	**14**
	**T02**	**3**	**4**	**4**	**2**	**3**	**4**
	**T02**	**8**	**4**	**12**	**6**	**7**	**6**
	**T05**	**0**	**0**	**0**	**0**	**0**	**0**
	**U62**	**0**	**0**	**0**	**0**	**0**	**1**
	**TOTAL**	**235**	**185**	**160**	**292**	**438**	**475**

To more closely examine the proteases present in SMBS, we looked at enzymes that were in highly represented families, and were transcripts with a high number of reads mapping to them (TPM) or had high similarity to proteases known from other parasite species. In particular, we examined cysteine proteases in the MEROPS family C01 – cathepsin L, aspartic proteases in the family A22 – Signal peptide and Presenilin-like proteases, metallopeptidases in the M12 – the metzincins, and S09 the Prolyl oligopeptidase family.

**Fig. 3:**
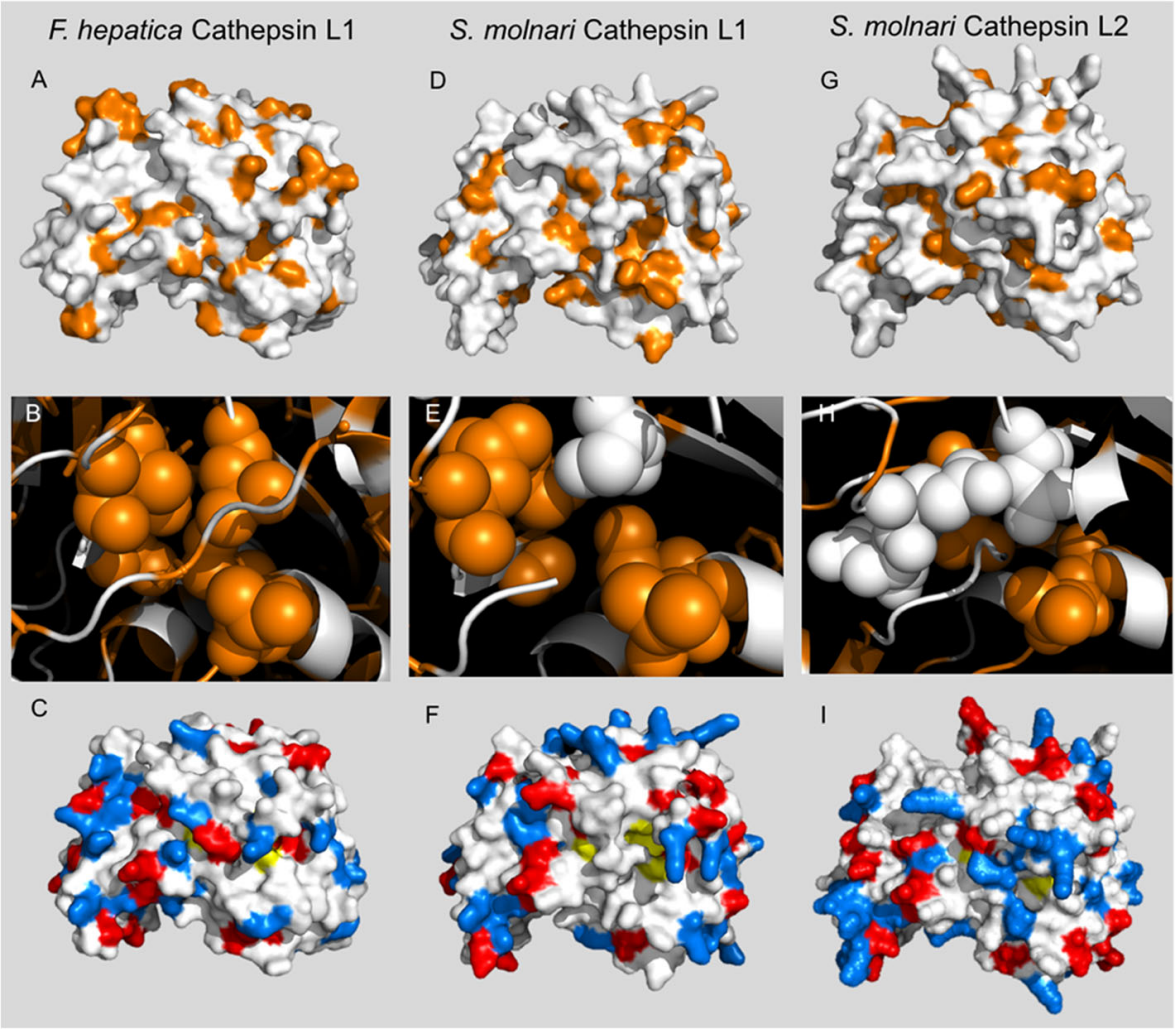
Predicted tertiary structure of SMBS Cathepsin L 1 and 2 and comparison with *Fasciola hepatica* cathepsin L. **a***Fasciola hepatica* cathepsin L crystalised structure 2O6X showing hydrophobic residues (orange). **b** Sm_CL 1 predicted structure based on Phyre2 model aligned to *F. hepatica* pdb. **c** Sm_CL2 predicted structure based on Phyre2 model aligned to *F. hepatica* pdb. **d-f** Closer view of substrate binding site 2, D - *F. hepatica* cathepsin, E – Sm_CL1, F – Sm_CL2. **g-i** Charged residues white = neutral, red = negative, blue = positive of G - *fasciola hepatica*, H - Sm_CL1, I - Sm_CL2

*Cathepsins: S. molnari*’s transcriptome revealed eight cathepsin-L like sequences by sequence homology, however, five were excluded from our further analysis due to 1) incomplete transcript and 2) missing or uncharacterised active sites at either substrate S1 or S2 sites, or 3) a sequence homology that appeared to be closest to cathepsin L but in fact had chymotrypsin-like folds (Ser-His-Asn) or Gly-His-Asn catalytic triads. Three cathepsin Ls analysed (CathL1–3) were all propeptides with signal peptides which may indicate later activation within the cell (e.g. lysosome) or extracellularly [32]. All had conserved a glutamine for the oxyanion hole known for cysteine peptidases, however, Sm_CL3 did not retain a stabilising asparagine close to the His active site, this was replaced by a negatively charged aspartic acid. We aligned the predicted tertiary structures of *S. molnari* cathepsin Ls to a procathepsin L from a metazoan parasite with x-ray crystallography evidence (PDB: 2O6X, [33]) (Fig. 3a-c). Two of *S. molnari*’s cathepsin Ls, Sm_CL 1 and 2 were able to be aligned to the crystal structure with high confidence, however, there was a marked difference in the distribution of hydrophobic resides (Fig. 3b-c). In particular, there were higher numbers of hydrophilic residues at the active site compared to *Fasciola hepatica* (Fig. 3d-f). The number of charged residues were similar overall, however the distribution of positive and negatively charged amino acids was different between all three proteins (Fig. 3g-i).

**Fig. 4:**
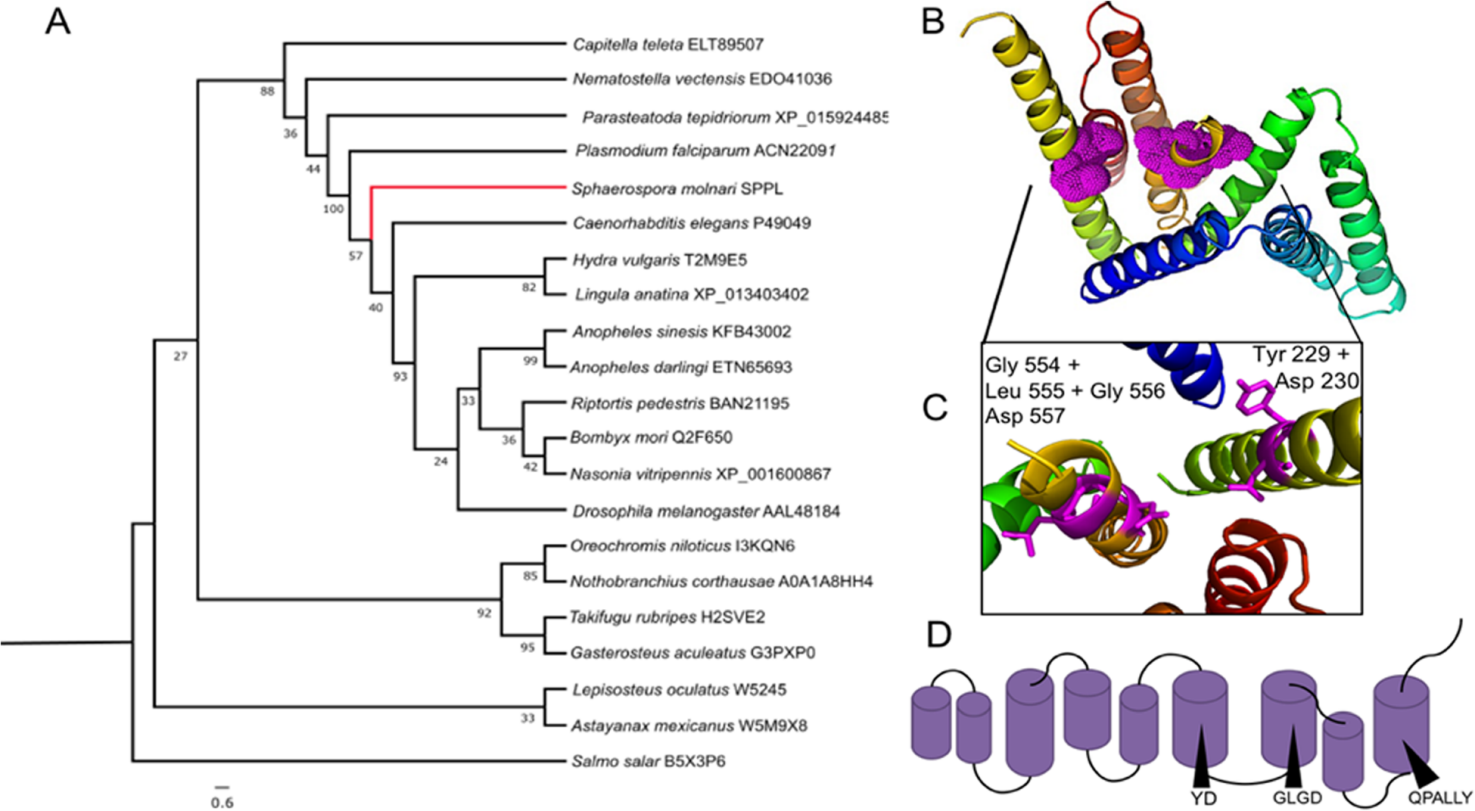
Aspartic protease of *S. molnari* – signal peptide peptidase. **a** Phylogenetic relationship of *S. molnari* SPPL (Sm_SPPL), (ML, 1000 bootstrap support values shown at nodes). **b** Predicted tertiary structure of Sm_SPPL according to Phyre2, showing nine transmembrane domains with active site highlighted in pink. **c** Closer view of active site and interacting residues. **d** Schematic of Sm_SPPL structure showing location of active sites and motifs within sequence

*Aspartic:* We identified two aspartic proteases in the transcriptome of SMBS, one presenilin-like and a signal peptide peptidase-like sequence (Sm_SPPL). The presenilin-like protease had a lower TPM value than Sm_SPPL (12.66272 compared to 146.829). We therefore focused our analysis on Sm_SPPL, which clustered with other invertebrates (Fig. 4a) and conformed to the structure of a signal peptide peptidase including active residues in transmembrane domains 6, 7 and 9 in the predicted model (Fig. 4b, c, d). Sm_SPPL has the appropriate active site residues and was able to align the predicted tertiary structure of a SPPL. Sm_SPPL was distinguished from a presenilin by the presence of QPALLY motif in its last transmembrane domain, like others in this group [34].

*Metallopeptidases:* Seventy two metallopeptidases in 16 of 29 families were flagged in the *S. molnari* proliferative stage peptidases. All of the *S. molnari* metallopeptidase families were shared by one or both of the other myxozoan species (*M. cerebralis* or *K. iwatai*) examined as well as two or three of the free living species (Table 3). The highest proportion of SMBS metallopeptidases were in the M12 family (adamalysins and reprolysins). We examine five of these metallopeptidases in more detail (Sm_MP1 TPM = 14.5; Sm_ MP2 TPM = 16.17; Sm_MP3 TPM = 22.07; Sm_MP4 TPM = 2.76; Sm_MP5 TPM = 0.79). All except one of the five have the methionine turn and zinc binding motif HEXXHXXGXXH with either a serine or an aspargine binding residue. Two of the target metallopeptidases had signal peptides, and almost all had reprolysin and disintegrin domains. Sm_MP4 has a potential C-terminal transmembrane tail that may anchor it similarly to other well-known ADAM proteins.

**Fig. 5:**
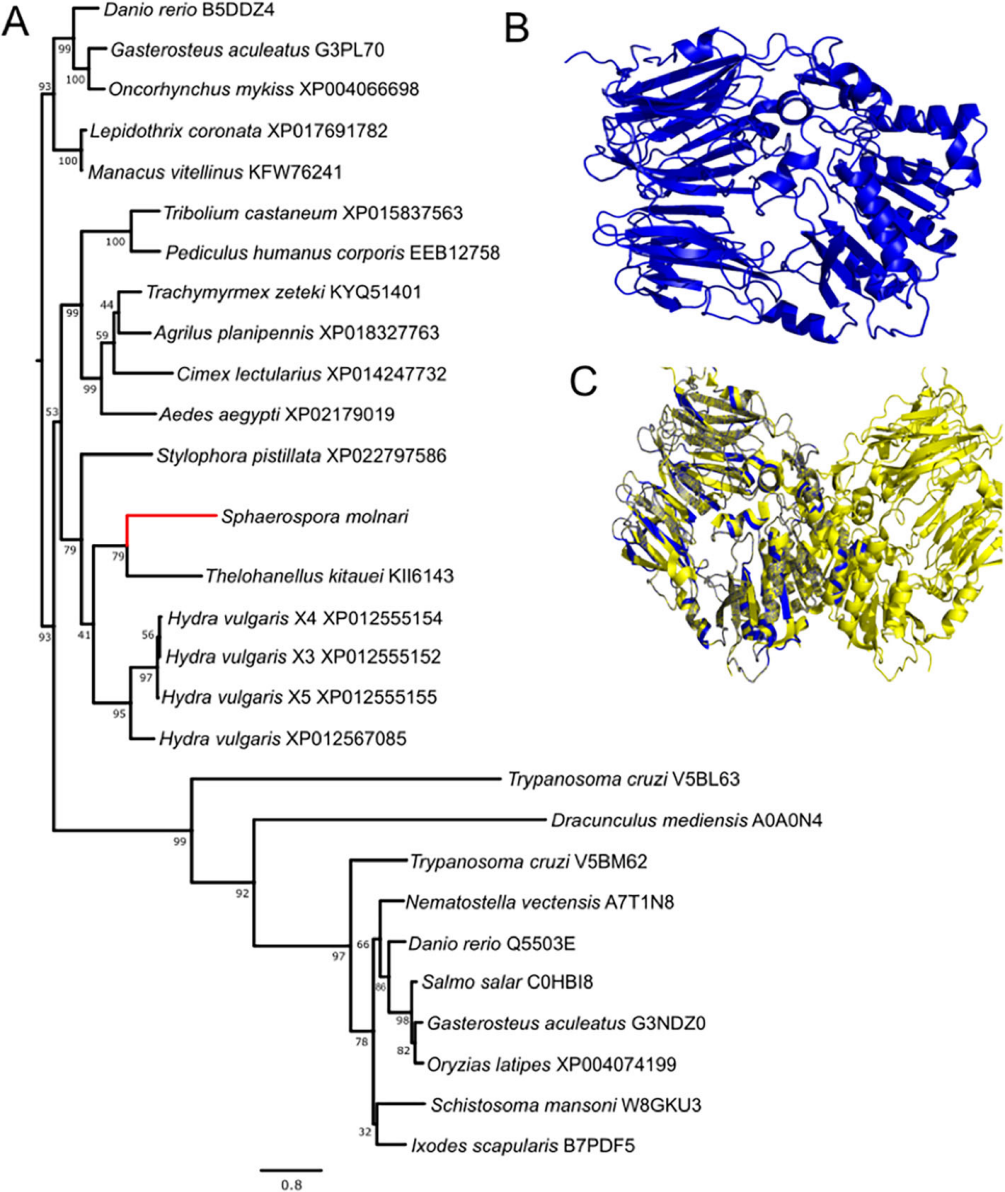
Phylogenetic placement and predictive structure of *S. molnari* dipeptidyl peptidase. **a** maximum likelihood phylogenetic analysis of *S. molnari* serine protease (MEROPS family S09) showing grouping with dipeptidyl peptidases rather than prolyl oligopeptidases with *Thelohanellus kitauei*. **b** Predictive structure of Sm_DPP as a monomer. **c** Overlayed predictive structures of human dipeptidyl peptidase IV dimer (pdb id: 2G5T, yellow) and Sm_DPP (blue)

*Serine:* Multiple serine proteases were identified within SMBS transcriptome, the largest was MEROPS family S09. The transcriptome of SMBS yielded a serine protease with sequence and structural homology to dipeptidyl peptidase 4 (Fig. 5b, c). SMBS_DPP contained the catalytic triad (Ser-633, Asp-711, His-743), a predicted transmembrane domain at the N-terminus. Phyre2 was able to model the sequence with 100% confidence according to the models of other dipeptidyl proteases (DPPIV and VIIII). The sequence clustered to *Thelohanellus kitauei* DPPIV and *Hydra vulgaris* POP isoforms (Fig. 5a) however, both of the *H. vulgaris* isoforms contained a transmembrane domain and a high homology to DPPIVs rather than POPs.

**Fig. 6:**
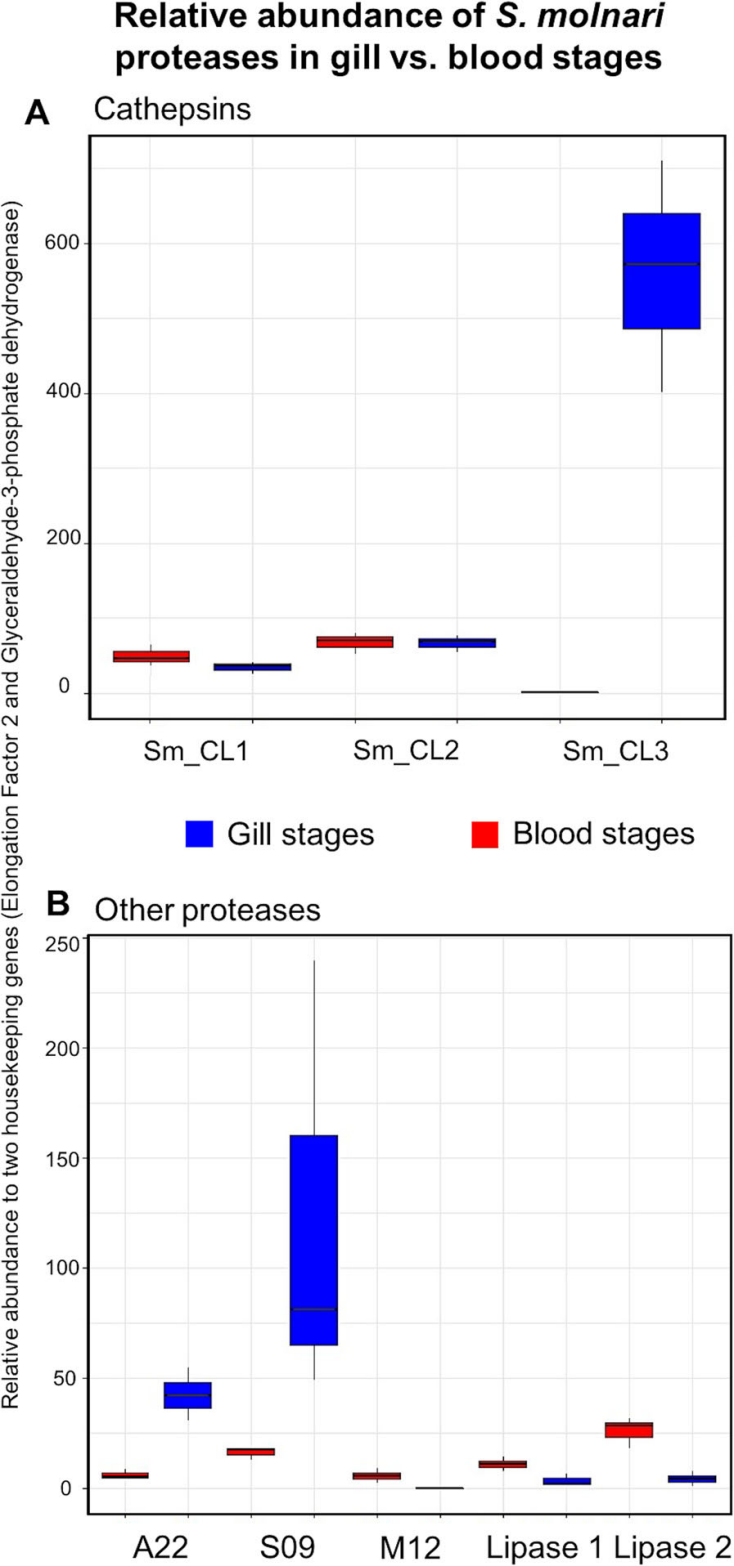
Real-time PCR of selected proteases in *S. molnari* blood stages (non-sporogonic) and gill stages (sporogonic). Relative abundance to two housekeeping genes (Elongation Factor 2 and Glyceraldehyde-3-phosphate dehydrogenase) in cDNA samples of circulating blood stages (n = 3) and spore forming gill stages in common carp (*n* = 3) including 95% confidence intervals and average for each marker. **a** Cathepsins (Sm_CL1, 2 and 3) in blood stages (red) and sporogonic gill stages (blue); **b** Other proteases (A22 – Signal peptide and Presenilin like peptidases (Sm_SP1); M12 metallopeptidase (Sm_MP1); S09 – Prolyl oligopeptidase (Sm_DPPIV 1) and Lipases (Sm_Lipase 1 and 2) in blood stages (red) and sporogonic gill stages (blue)

We then examined the expression of eight key proteases in blood stages compared to spore forming gill stages by qPCR and also in silico expression (TPM). Three cathepsins Sm_CL1, 2 and 3; a presenilin like aspartic protease (Sm_SP1); a dipeptidyl peptidase (Sm_DDPIV); a metallopeptidase (Sm_MP1) and two lipases (Sm_Lip1 and 2) were used as target proteases. Half of our candidates were upregulated in blood stages rather than gill stages (Fig. 6). Expression of Sm_CL3 was over 500x higher in sporogonic gill stages than presporogonic SMBS (Fig. 6a). Sm_CL3 was predicted to contain a transmembrane domain and had a relatively low TPM value according to our assembly (2.3503) compared to the other two cathepsins Sm_CL1 and 2 (TPM = 782.87 and 77.28, respectively), both of which had signal peptides and were transcribed as procathepsins. Both Sm_CL1 and 2 showed similar expression in the blood and the gills. An astacin metallopeptidase and two lipases were also upregulated in blood compared to gill samples (Fig. 6b). In contrast, both the aspartic and serine proteases, Sm_SP1 and Sm_DPPIV respectively were significantly upregulated in spore forming stages in the gills. Sm_DPPIV did not have a high relative expression within our transcriptome (TPM = 3.99), however when we compared its expression in blood stages vs. gill stages, we found it was more highly expressed in sporogonic gill stages by almost 100 fold. Sm_SP1 had a higher TPM value than Sm_DPPIV (146.829) and was 42 times more highly expressed in the gills than blood.

## Discussion

This transcriptome analysis of *S. molnari* is the first from this entire clade of sphaerosporid myxozoans, this is the only group for which an invertebrate host is unknown and is the only dataset from an extrasporogonic stage of development. Therefore, it offers a unique insight into the mechanisms of myxozoan development and host interactions. Our focus on proteases, was primarily in the pursuit of identifying proteins that would be worthy of further investigation to understand their role in the host-parasite relationship.

The transcriptome of *S. molnari* blood stages (SMBS) appears to follow a similar trend as other parasitic and free living cnidarians in its heavy AT richness for many of its genes [35] and its overall reduction in genes [11, 12]. Gene divergence and AT richness of the SMBS dataset aids distinction of host and parasite genes in many cases and could also aid targeted gene therapy as seen in other anti-parasite drug design e.g. *Plasmodium falciparum* [36]. The number of proteins identified could have been limited due to the extensive host filtering we conducted on the dataset, divergent genes and/or the nature of the particular life stage of SMBS. Separation of the SMBS from the host immune cells would significantly improve further transcriptomic analysis to combine physical and bioinformatic filtering. More than half of the known BUSCO metazoan genes were identified in this dataset, similarly to *M. cerebralis* triactinomyxon stages and sporogonic *K. iwatai* transcriptomes. Although using a different comparative dataset (CEGMA, previous benchmarking metazoan dataset) Chang et al. [11] also noted a reduced number of core eukaryotic genes in the transcriptomes and genomes of *K. iwatai* (70% of CEGS, compared to our 56% of BUSCOs) and *M. cerebralis* (39% of CEGS compared to 55% of BUSCOs) whilst *P. hydriforme* consistently had more than its myxozoan relatives and was closer in numbers to the free living species (90% of CEGs and 89% of BUSCOs). BUSCO genes are also used to estimate the completeness of a genomic or transcriptomic dataset, whilst it is unlikely that we sequenced the extent of the expressed transcripts of *S. molnari* due to overwhelming host contamination, it is interesting to note an overall reduction in this gene set for myxozoans in general. For comparison, other early branching metazoans and cnidarians have retained a high percentage of these conserved proteins, e.g. Placozoa species *Hoilungia hongkongensis* had 90–95.3% [37]; sponge *Halisarca caerulea* had 92.6% [38]. Free living cnidarians have variable accounts of their retained complete BUSCO genes, e.g. *Acropora digitifera* 69% (675/978) [39]; *Exaiptasia pallida* 84.5% (826/978) [39]; Scleractinian coral *Porites lutea* 56.4% [40]. Other metazoan parasites have also retained a higher level of genes e.g. the trematode *Microphallus* sp. maintained close to 70% of genes from the same metazoan dataset [41]. The transcriptomes of parasitic nematode *Teladorsagia circumcincta* were found to have only 23–38% of the conserved gene set yet appeared to have good matches to the representative genome [42]. Furthermore, cestode parasites have a range of conserved eukaryotic genes from *Taenia multiceps* (81.8%) to *Echinococcus multilocularis* (91.6%) [43]. Additionally, despite removing protein sequences that were more than 90% similar to each other in the *S. molnari* dataset, there were a high number of duplicated single copy BUSCO genes (Table 2). This may be caused by the pooling of wild-type individuals sampled from different locations and populations. De novo assemblers such as Trinity, designed to generate alternative transcripts, are more sensitive to sequencing errors and/or highly heterozygous datasets, and thus multiple loci may have been assembled into ‘isoforms’ of the same parent transcript increasing the amount of duplication [44, 45].

We examined some specific examples of proteases of interest within the *S. molnari* transcriptome that were homologous to other known parasitic proteases involved in important host-parasite functions. There are examples of parasitic proteases that have been exploited as drug targets in all different groups and protease families. Two of the aspartic peptidases we identified in the SMBS transcriptome were also in groups that had been identified as potential drug targets. A presenilin-like and a signal peptide peptidase-like protease (Sm_SPPL) were found in SMBS both are in the MEROPS protease family A22. The proteases are closely related, SPPLs are transmembrane proteases with their catalytic sites buried in bordering transmembrane domains, and have varied functions in eukaryotes depending on their localization within the cell [46, 47]. In humans, they are involved in the processing of peptides for the MHC I epitopes and their central role in processing signal peptides indicates an important role in signaling and protein modification as part of the endoplasmic reticulum [34]. In contrast to SPPLs, presenilins require a co-factor and multiple subunits to form a catalytic γ -secretase complex [46]. Both aspartic proteases have been identified as potential drug targets in *Plasmodium falciparum* [48], and can potentially play a role in the cleavage of peptides for antigen presentation on a cell surface [49]. SPPL blockers have been shown experimentally in parasite-derived homologs e.g. LY411,575 (reduced *Plasmodium berghei* in mice and humans [50]), and bioinformatically as suggested in protozoan species *Toxoplasma gondii*, *Leishmania infantum*, and *Trypanosoma cruzi* [51]. Both *S. molnari* proteases are divergent from their hosts and in the case of Sm_SPPL have been shown to be highly expressed during its sporogenesis development in the host’s gills. This localization may be useful for targeting future therapeutic assays at such an aspartic protease in this system.

Another group of proteases that are commonly targeted for anti-parasite therapy are the metallopeptidases. These have been associated with many functions of parasitic development, they are linked to digestion e.g. falcilysin in *Plasmodium falciparum* [52], host immune system disruption e.g. matrix and secreted metalloproteases [1, 53]; or host immune system evasion e.g. Leishmania GP63 [2]. SMBS had a large variety of metallopeptidases, it is also important to note families that are missing in the myxozoan species, for example we did not identify any M08 homologues which have been originally characterised in *Leishmania* to aid immune system avoidance in promastigotes [54]. Nor did we identify any matrix metallopeptidases (M10 family) which have also been previously identified as protozoan parasite drug targets [55, 56]. One of the largest groups we identified in SMBS was M12, the metzincins. These have been found in other parasitic organisms feeding in their host’s blood and are considered important for digestion or nutrient uptake which may be their role in SMBS as they are circulating in the host’s blood. The diversity of SMBS metzincins also indicates varied roles for these metallopeptidases in these developmental stages. Although the function of each specific protease are not known, and they were not considered to be highly expressed (TPM values range from 0.7–22.07), they share some valuable attributes that make them strong candidates for future research. Primarily, they share a key domain and binding site and none of the potential target sequences (Sm_MP1–5) had “cysteine rich” domains identified in their sequence which has been discussed as a possible hindrance to recombinant protein production [57]. Recombinant protein application or other interference with parasite metzincins has been shown to be a successful anti-tick treatment [58] and may potentially be employed with any of these SMBS metallopeptidases. A recombinant protein with epitopes of multiple SMBS metzincins, considering they are so divergent from each other as well as any recognisable fish host homolog, could hold potential for a therapeutic experiment.

In the serine proteases, the largest family found in SMBS was S09. This family has multiple groups of which only some have been identified as putative drug targets. One such group is the dipeptidyl peptidases (DPPs) and prolyl oligopeptidases (POPs), which specialize in the cleavage of proline residues (DPPs cleave at N-terminus, POPs cleave at C-terminal). The S09 protease we looked at as a potential drug target in SMBS had similarities to both groups however we determined it to be a DPP according to its sequence, phylogenetic position and its structural homology to DPPIV (Fig. 5). DPPIV has also been identified in bee and snake venoms and possibly influences vasodilation and constriction, something that may be useful to SMBS in host blood vessels. DPPIV exists as a homodimer (potentially tetramer) targets proline residues and has suggested roles in antigen presentation on the cell surface, cell adhesion and collagen binding activity [59]. In protozoans, DPPIVs have been shown to play a role in the encystment of *Giardia*, a process that can be blocked by the application of inhibitors [60]. DDPIVs have been associated and inhibited in other blood-feeding parasites including *Haemonchus contortus* where it is suspected to play a role in fibrogen breakdown and coagulation in the blood [61]. Due to the choice and variety of DPPIV inhibitors that are available for both human and veterinary medical applications, we propose that SMBS DPPIV could be a good target for assessing the impact of its inhibition on the proliferation and metabolism of *S. molnari* in its host. This is based partially on its unique sequence as evidenced from its relation to other homologs, its predicted structure which could aid the application of known inhibitors, and its expression in a developmental stage that could be feeding on blood.

One of the most well researched protease groups in parasites are cysteine proteases, commonly investigated for their potential as therapeutic targets in parasites as they are known to be involved in a variety of pathways as well as being associated with parasitic development and proliferation [62]. In myxozoans, cysteine proteases have been suggested to be involved in the proteolytic destruction of host tissue based on activity and substrate assays [10, 63, 64]. They are also associated with host hemoglobin degradation in blood feeding parasites, e.g. many parasitic helminths [65] and maybe the case for *S. molnari* which proliferates in host blood and interacts with erythrocytes [29]. The replacement of a stabilising asparagine close to the His active site of Sm_CL3 was also seen in a cathepsin L of the myxozoan *Kudoa thyrsites. K. thrysites* is suggested to use its cathepsins for the degradation of host tissue, i.e. “Milky Flesh Disease” [9]. *S. molnari* cathepsins appear to have all of their disulfide bridges in the three isoforms we examined in contrast to the cathepsin Ls reported from *K. thyrsites* [9]. Sm_CL1, 2 and 3 had close homology to *Fasciola hepatica* cathepsins, these proteases are expressed throughout *F. hepatica*’s development with distinct roles in feeding and invasion based on their binding sites [62, 66]. All Sm_CLs had higher numbers of hydrophilic residues at the active site compared to *F. hepatica* (Fig. 3a-c). The number of charged residues were similar to each other overall, however the distribution of positive and negatively charged amino acid was different between all three proteins (Fig. 3). These changes could potentially impact the activity and substrate affinity for the proteases. Cysteine proteases are particularly studied in parasites due to their roles in moulting, encysting and digestion across parasite taxa, and in particular they are often targeted for anti-parasite therapies or inhibition [62, 65, 67–69]. Here, we present the first expression analysis of cathepsins in SMBS that are potentially important in the feeding, immune evasion or tissue penetration of this parasite in its host. Characterisation of these proteases (substrate, inhibitors, abundance in the proteome) will advance our knowledge of the roles these cathepsins play in the development of this parasite and further inform our prioritization of protease targets for intervention and control assays.

Cathepsin Ls were the most highly expressed (using TPM as indication) proteases identified within our transcriptome, that combined with the large number of in vitro and in vivo experiments on other parasite cathepsins and their effect on parasite survival make them prime candidates for further development. Some examples use peptides mimicking compounds such as aziridine-2,3-dicarboxylate-based inhibitors to inhibit cathepsin L activity e.g. *Trypansoma brucei rhodesiense* [4] or non-peptidic inhibitors e.g. chalcones in *F. hepatica* [70]. *S. molnari* is unusual in the number of cathepsin L’s expressed in its blood stages, not just isoforms but distinct proteases. Until in vitro assays are able to better pinpoint which cathepsins are linked to SMBS survival, we rely on sequence similarity and predictive modeling to infer homology to other cathepsins including those that have been inhibited successfully. Sm_CL1 and 2 as described above, had signal peptides for potential secretion and they differed in amino acid composition (37.0% identity). Sm_CL2 had more hydrophobic residues at the S1 active site and Sm_CL1 was more highly expressed in the blood stage than Sm_CL2. Both had predicted structures that aligned to FhCL1 *F. hepatica*’s cathepsin’s structure which has been inhibited as well as another of its cathepsins FhCL3 with flavonoid compounds both experimentally and with computational docking [70]. Both *F. hepatica* cathepsins exhibited hydrophobic interactions with compound C34, and were successfully inhibited although at different rates depending on the concentration of the compound. Experimental evidence would be needed, however we propose Sm_CL1 and 2 as good candidates for inhibition with flavonoids such as C34 in future in vitro assays. The divergence in sequence identity from their host, their expression in both blood and gill stages and their similarity to proteases that have been successfully blocked in such assays are all good evidence that these could be the first drug targets for *S. molnari.*

Comparison of the expression of some of the proteases in non-spore forming blood stages and gill sporogonic stages gave further insights into the function of some groups. The cathepsin Ls that we examined each had a different expression profile during SMBS development, with Sm_CL3 being the main cathepsin expressed in the gills. Localisation and expression would influence which cathepsin to target for future work, and may indicate roles in sporogenesis for Sm_CL3 compared to CL1 and CL2 in the blood. CL1 and 2 may act as other cathepsins in blood feeding parasites and break down hemoglobin for sustenance. Lipases and metallopeptidases are also associated with feeding in parasites and were comparatively higher in SMBS than in the gills. Astacin metallopeptidases have also been shown to break down complex proteins such as hemoglobin or migrate through tissues [4, 71, 72], and its upregulation in the proliferative developmental stage rather than spore forming could indicate a role in nutrient acquisition or penetration of target tissues (Fig. 1) in SMBS. Lipases have been suggested to play role in parasite cell surface protein presentation as well as digestion of host protein [73, 74], the two candidates we show are upregulated in SMBS should be further investigated for their substrate and localisation within the SMBS to further characterise if they are integral to parasite survival. Disruption of such enzymes could be of interest to uncover the proteomic arsenal SMBS use to digest and navigate its host for survival. Conversely, the aspartic (Sm_SP1) and serine protease (Sm_DPPIV) would be more informative to investigate where they appear to be highly expressed – the gills. These two proteases have been associated with antigen presentation and host immune system avoidance, it could be significant that they are relatively abundant when the parasite is bound within host target tissue, rather than its extracellular blood stage, where it would be directly in conflict with the host immune system. Localisation and further characterisation will be vital to learning more about the role these proteases play in the developmental stages we identified them in and determine if they are interesting targets with anti-parasitic potential.

## Conclusions

We produced and explored the first transcriptomic dataset of early proliferative myxozoan stages to date and identified family expansions in cysteine, metallo and threonine clans. We did not identify any myxozoan-specific radiations in particular groups, however, all of the myxozoan proteases we examined were highly divergent from each other as well as from other cnidarians. Vaccine development against a number of metazoan parasites is based on proteases as antigens of interest [75–77]. However, with regard to myxozoans, the function and involvement of these enzymes in host-parasite interaction first need to be elucidated as a major lack of knowledge exists with regard to metabolomics and the molecular means of host interaction. The general strategy for therapeutically targeting proteases is to identify a specific inhibitor — generally a small molecule — that blocks the active site. Discovery efforts for new inhibitors have typically been based on the structure of known protease substrates, presenting a substantial challenge for the development of peptidomimetic compounds that have the pharmacokinetic characteristics needed to be suitable as a drug.

This study advances our knowledge of myxozoan protease sequence, predicted structure and in some cases hydrophobicity and amino acid changes. This information furthers investigation into the potential role these proteases play in the development, sustenance and host immune evasion of these important parasite stages. Vaccination plays an important role in commercial large-scale fish farming and is a key reason for the success of salmon aquaculture, however, available vaccines are aimed at bacterial and viral pathogens, while parasite vaccines for fish are still inexistent [78], likely due to an insufficient knowledge of potential parasite target molecules of fish parasites, when compared with parasites of human or veterinary importance. It is now more than timely to explore new genomic data for such targets as epidemical models predict major emerging disease outbreaks and an increased geographic range of myxozoan species such as *T. bryosalmonae*, in relation to temperature increase [79, 80], with new records from northern European territories [81–83] and recent reports of massive fish killings from the Yellowstone river [84, 85], Hutchins et al. 2017). Exploring the enzymes expressed during early establishment and proliferation of myxozoan infections is essential to finding putatively relevant vaccine targets that can inhibit rapid multiplication of cryptic parasite stages in fish, long before the onset of disease. The proteases we discuss here are putative targets for further research, confirmation of their expression in different stages of *S. molnari*’s life cycle (SMBS vs. gill sporogonic stages vs. extracellular secretion) would be an invaluable method of testing their activity and function and therefore their use in anti-parasitic development.

## Methods

### Animal and sample collection for transcriptome analysis

Common Carp (*Cyprinus carpio*) were obtained from two localities, Štrmilov in Czech Republic (49.1644° N, 15.2031° E) and Hortobágy in Hungary (47.3542° N, 21.0000° E) during 2013–2015. All fish were obtained commercially, were less than 2 years old and were transported live to the laboratory. Fish were anaesthetized with clove oil and blood was taken, all animal procedures were performed in accordance with Czech legislation (section 29 of Protection of Animals Against Cruelty Act No. 246/1992) and approved by the Czech Ministry of Agriculture. 8 fish were found to be infected with *Sphaerospora molnari* blood stages (SMBS), and whole blood was centrifuged for 5 min at 3500 rpm in heparinized hematocrit tubes to isolate host white blood cells mixed with SMBS.

### For qPCR analysis

Presporogonic *S. molnari* blood stages and sporogonic gill stages were isolated from experimentally infected fish (*n* = 3) in the Czech Republic or recirculation system in Hungary (*n* = 3). Specific parasite free fish tissues were selected from laboratory cultures within the Czech Republic (*n* = 3). Fish were euthanized according to the ethics license and methods above.

### RNA isolation and Transcriptome assembly

Total RNA was isolated from blood stages/host white blood cell mixtures with Macherey-Nagel NucleoSpin Kit II (Biotech, Czech Republic) for transcriptome sequencing at Beijing Genomics Institute (Bejing Genomics Institute, Hong Kong) with an Illumina HiSeq 2000 (75 bp paired end reads). Reads were filtered for bacterial contaminants and then aligned to the Common carp genome (ftp://ftp.ncbi.nlm.nih.gov/genomes/all/GCA/001/270/105/GCA_001270105.1_ASM127010v1) with bowtie2, using -very-sensitive parameter. All remaining reads were then assembled with Trinity v2.4.0 and the transcripts were compared with the carp genome again for further host transcript identification (BLAST parameters: tblastx, 1e^− 05^), those with a percentage identity of > 75% were removed to create a “non-host” dataset which was translated to protein using OrfPredictor [86]. Redundancy of the translated non-host dataset was removed with CD-Hit using 0.9 cutoff [87] and blastp (1e^− 05^) annotated with NCBI nr database.

### Annotation

The non-redundant dataset was screened for assembly quality and completeness by identifying BUSCO genes (http://busco.ezlab.org/) using the metazoan 09 dataset [31]. Proteases and inhibitors were identified using the “meropsscan” dataset (https://www.ebi.ac.uk/merops/index.shtml) using blastp, 1e^−^ 05 [88]. Signal peptides and transmembrane domains were predicted using Signal P and TmHmm (http://www.cbs.dtu.dk/services/).

### Comparative assemblies

Transcriptomic sequences for other cnidarian species were downloaded from NCBI: Myxozoans – *Myxobolus cerebralis* triactinomyxon stages from *Tubifex* host (PRJNA258474), *Kudoa iwatai* myxospores from cysts of *Sparus aurata* tissue (PRJNA248713). Non-myxozoan cnidarians with endoparasitic life stages - *Polypodium hydriforme* non-parasitic stolons (PRJNA251648), and *Edwardsiella lineata* (mix of parasitic and free living life stages) (downloaded from EdwardBase site when was active in 2014 [89]. Finally, to compare with a completely free living relative, genome derived RefSeq mRNA sequences of the anthozoan *Nematostella vectensis* PRJNA19965 were downloaded from NCBI. Transcripts were translated into peptide sequences by OrfFinder [86] and searched for proteases as above, the redundancy was removed (0.9 cutoff) and the non redundant dataset was screened for BUSCO genes as above.

*Target protease groups*: Four groups of proteases were more closely examined (A22 – Signal peptide peptidases and presenilins, C01-Cathepsin Ls, M12 – metalloendopeptidases, S09 – Prolyl oligopeptidases and dipeptidyl peptidases). Representative *S. molnari* sequences with other sequences from Genbank and Uniprot (including fish sequences) were analysed phylogenetically with fish and other cnidarian and parasitic-derived sequences using RaxML (L + G + I). Tertiary structures were predicted for key proteases using the Phyre2 server [90] and models were compared and manipulated in PyMol ver. 1.4.1 (The PyMOL Molecular Graphic System).

#### Sanger sequencing of key predicted proteases and rDNA

RNA was extracted from 3 biological replicates of *S. molnari* proliferative blood stages (pooled samples from several individuals) and spore-forming stages (individual fish). Total host+parasite RNA was isolated using the Nucleospin RNA Kit (Machery-Nagel) including a DNase treatment step. RNA concentration and purity was checked using a Nanodrop (ND-1000) Spectrophotometer (NanoDrop Technologies) and cDNA was synthesised using the Transcriptor High Fidelity cDNA synthesis Kit (Roche). Primers were designed to amplify full length sequences of selected proteases and ribosomal DNA as single amplicons or with long overlaps between individual sections, to confirm the assembled transcriptome sequences.

#### Quantification of stage-specific expression of candidate proteases

Gene-specific primers were designed to amplify short, 70–150 bp regions suitable for qPCR (Supplementary Data). All primers were tested for functionality and specificity using conventional PCR prior to performing qPCR. qPCR was performed using the FastStart Universal Sybr Green Master Mix (Rox) on LightCycler® 480 Real-Time PCR System (Roche). Reactions contained 12.5 ul of FastStart Universal SYBR Green PCR Master Mix, Roche, Germany (2X conc.), 1 μl of each forward and reverse primer (10 μM conc.), 5.5 μl of PCR grade water, and approx. 500 ng of cDNA, resulting in a final volume of 25 ul. Cycling conditions were as follows: Denaturation at 95 °C for 5 min, followed by 50 cycles of 95 °C for 10 s, and 58 °C for 10 s and 72 °C for 10 s. Melting curve analysis were performed after each qRT-PCR to ensure primer specificity. The relative expression ratio of each sample was calculated according to Pfaffl [91], based on the take-off deviation of sample versus controls at each time point and normalized relative to Elongation Factor 2 and Glyceraldehyde-3-phosphate dehydrogenase (housekeeping genes, [29]). Confidence intervals, and box plots made in R.

## Supplementary information

**Additional file1.** Supplementary Data 1: Table of primers used in qPCR assay including house keeping genes.

## Data Availability

Raw unfiltered sequence reads are deposited to Genbank under Bioproject PRJNA522909. Primers used for qPCR are provided as supplementary material. Host and parasite transcripts are available through Dryad depository link Sanger sequenced ribosomal DNA was submitted to Genbank under MK533682.

## Abbreviations

SMBS: *Sphaerospora molnari* Blood Stages
ORF: Open Reading Frame
BUSCO: Benchmarking Universal Single Copy Orthologs
CEGMA: Core Eukaryotic Genes Mapping Approach
Cp: Copy
TPM: Transcripts Per Kilobase Million
DPP: Dipeptidyl peptidases
POP: Prolyl oligopeptidases
