# Supplementary material for: Transcriptome of Sphaerospora molnari (Cnidaria, Myxosporea) blood stages provides proteolytic arsenal as potential therapeutic targets against sphaerosporosis in common carp
Source: BMC Genomics. 2020 Jun 16;21:404. doi: 10.1186/s12864-020-6705-y (PMC7296530; doi:10.1186/s12864-020-6705-y)
Supplement: Supplementary file 1 — Additional file1. Supplementary Data 1: Table of primers used in qPCR assay including house keeping genes. [file 12864_2020_6705_MOESM1_ESM.pdf]

Supp. Data. Primers used for comparative protease expression studies of *S. molnari* pre-sporogonic vs sporogonic stages using qPCR .

Primer ID	Amplified gene name	Primer sequence (5'-3')	Amplicon length (bp)
Sm_SP-1	Presenilin like (A22 family)	F: GTTTCGGAAGGGTCTGGAGG<br>R: GCAAAGACGAGCAAGAGCTG	123
Sm_CL3	Cathepsin L (C01 family)	F: TACGGTTGTTGGAGTCACCG<br>R: TTGCCCCTGAAGCAATTCCA	128
Sm_CL1	Cathepsin L (C01 family)	F: TGTCGACATCAAAAACAGCAGA<br>R: CGAGAGATCCAATCGCACTG	150
Sm_CL2	Cathepsin L (C01 family)	F: ATGCTTGCAAGAGTGGTCGA<br>R: GTGAGGCTCAGGTTCGAGAC	171
Sm_EF2	Elongation Factor 2	F: TCCGGCAGGCAAGAAGGTTT<br>R: CCAAGTTGGATACGGATTACGAGT	140
Sm_GAPDH	Glyceraldehyde-3-phosphate dehydrogenase	F: TATCGACCTGGCCGTTACTG<br>R: GTTGCTGCTGTCAATGACCC	118
Sm_Lipase 1	Lipase	F: TTTGTGTAGGACAGCGTGGT<br>R: AGCTCGAGTTTCCTCTGTGC	108
Sm_Lipase 2	Lipase	F: GCGAATTTCGACCTTCCACA<br>R: AGGTTCACTTCCCCATGCTG	120
Sm_Metallopep1	Astacin metallopeptidase	F: ACCCTGAAGGCTGGTGTAGA<br>R: GTTCCTTTCTCGCGGCATTC	119
Sm_DPPIV 1	Dipeptidyl peptidase	F: TTGCGCAATCCAGGACTTCT<br>R: ACAAGACACTTCCGTGGGTG	114
